# Low‐level laser therapy 810‐nm up‐regulates macrophage secretion of neurotrophic factors via PKA‐CREB and promotes neuronal axon regeneration in vitro

**DOI:** 10.1111/jcmm.14756

**Published:** 2019-10-31

**Authors:** Jiawei Zhang, Jiakai Sun, Qiao Zheng, Xueyu Hu, Zhe Wang, Zhuowen Liang, Kun Li, Jiwei Song, Tan Ding, Xuefeng Shen, Jianxin Zhang, Lin Qiao

**Affiliations:** ^1^ Department of Orthopedics Xijing Hospital, Fourth Military Medical University Xi'an China; ^2^ Department of Occupational and Environmental Health and the Ministry of Education Key Lab of Hazard Assessment and Control in Special Operational Environment Fourth Military Medical University Xi'an China; ^3^ Department of Orthopedics Weinan Central Hospital Weinan China; ^4^ Department of Orthopedics Third Hospital of Chinese PLA Baoji China

**Keywords:** axonal regeneration, low‐level laser therapy, M1 macrophage, macrophage polarization, spinal cord injury

## Abstract

Macrophages play key roles in the secondary injury stage of spinal cord injury (SCI). M1 macrophages occupy the lesion area and secrete high levels of inflammatory factors that hinder lesion repair, and M2 macrophages can secrete neurotrophic factors and promote axonal regeneration. The regulation of macrophage secretion after SCI is critical for injury repair. Low‐level laser therapy (810‐nm) (LLLT) can boost functional rehabilitation in rats after SCI; however, the mechanisms remain unclear. To explore this issue, we established an in vitro model of low‐level laser irradiation of M1 macrophages, and the effects of LLLT on M1 macrophage polarization and neurotrophic factor secretion and the related mechanisms were investigated. The results showed that LLLT irradiation decreased the expression of M1 macrophage‐specific markers, and increased the expression of M2 macrophage‐specific markers. Through forward and reverse experiments, we verified that LLLT can promote the secretion of various neurotrophic factors by activating the PKA‐CREB pathway in macrophages and finally promote the regeneration of axons. Accordingly, LLLT may be an effective therapeutic approach for SCI with clinical application prospects.

## 1 INTRODUCTION

Spinal cord injury (SCI) is a common affliction of the central nervous system. As result of its high incidence and poor prognosis, SCI has been a major challenge for the medical community.^1^ The regeneration of central nervous system axons after SCI is problematic and represents the main bottleneck in patient recovery. SCI can be divided according to the pathological stage into primary and secondary injury stages.^2^ The initial stage encompasses damage directly caused by the physical injury itself, which is mostly irreversible. However, the composition of the local physiological microenvironment of the injury site in the secondary stage differs from patient to patient, greatly affecting the individual prognosis.^3^

After SCI, both microglia and macrophages involved in the inflammatory response.^4^ However, microglia cells only play a role in the initial stage when they are involved in the clearance of damaged nerve fragments. While the peripheral macrophages, which were differentiated from blood‐derived monocytes massively recruited in the damage area, are the main causes of the further aggravation of the damage in the secondary injury stage.^5^ Macrophages are the most important components of the physiological microenvironment at the injury site during the second stage and consequently play an important role in the progression and prognosis of SCI.^6^ Recent studies have found that different local microenvironments can elicit different macrophage polarization states and functional phenotypes. These phenotypes are typically understood to include the cytotoxic classically activated macrophages (CAMs), termed M1 macrophages, and the anti‐inflammatory, nerve growth‐promoting, alternatively activated macrophages, termed M2 macrophages.^7^ Large numbers of macrophages enter the lesion in the immediate period after SCI and are activated to become the M1 type. Consequently, they play a major role, secreting high levels of proinflammatory factors and promoting necrosis and apoptosis. The appearance of the M2 macrophages is comparatively late and short‐lived.^8^ The specific polarization of macrophages after SCI is therefore not conducive to the repair and regeneration of axons.

Neurotrophic factors such as brain‐derived neurotrophic factor (BDNF), nerve growth factor (NGF), ciliary neurotrophic factor (CNTF), and leukaemia inhibitory factor (LIF) comprise a family of proteins that promote the survival and growth of neurons.^9^ Many studies have shown that stimulation of the ERK‐cAMP response element‐binding (CREB) or protein kinase A (PKA)‐CREB pathway can promote the secretion of various neurotrophic factors. In contrast to the proinflammatory M1 macrophages, the M2 type is capable of secreting many types of neurotrophic factors.^10^, ^11^ This finding implies that modulation of the polarization state of the macrophages in the SCI lesion can be used to promote nerve repair, which has become a focus of research into SCI recovery. A number of approaches can be used to modify the polarization of macrophages and thereby increase the relative abundance of the M2 cell type, which would in turn increase the levels of secreted neurotrophic factors and effectively increase the growth of neuronal axons, ultimately promoting neuronal growth and repair.

Low‐level laser therapy (LLLT) denotes the biological use of low‐radiation laser sources and is applied to promote tissue repair as a noninvasive growth‐promoting treatment. Furthermore, LLLT has been broadly investigated in the field of SCI treatment and recovery. Relevant studies have shown that 810‐nm LLLT can promote the recovery of SCI in rats, effectively boosting the recovery of the locomotor ability, reducing scar formation, significantly improving neuronal survival, modulating the inflammatory lesion microenvironment, suppressing the secretion of proinflammatory factors, and promoting the release of inflammation‐resolving factors.^12^, ^13^ In addition, LLLT improves the polarization state of the macrophages within the lesion site, reducing the relative abundance of the cytotoxic M1 macrophages while also increasing the proportion of nerve‐repairing M2 macrophages.^12^ However, whether LLLT can improve the secretion of neurotrophic factors by macrophages or promote the regrowth of neuronal axons is presently unknown, as well as the mechanism underlying such effects. Starting from these questions, we developed the experiments described in this manuscript to investigate the modulatory effects of LLLT on the secretion of neurotrophic factors by M1 macrophages as well as the underlying molecular mechanisms.

## 2 MATERIALS AND METHODS

### 2.1 Ethical statement

The present study was approved by the Institutional Animal Care and Use Committee of the Fourth Military Medical University (Xi'an, China) and was performed according to the principles of the National Institutes of Health Guide for the Care and Use of Laboratory Animals (National Institutes of Health, Bethesda, MD, USA).

### 2.2 Animals

BALB/c mice (6‐ to 8‐week‐old females and 1‐ to 3‐day‐old male and female juveniles) of the same strain were obtained from the animal facility of the Fourth Military Medical University (Xi'an, China).

### 2.3 Cultivation of dorsal root ganglion (DRG) neurons

Five juvenile BALB/c mice (1‐3 days old) were individually sacrificed in 75% ethanol at −20°C. The DRGs were extracted and placed in Dulbecco's modified Eagle's medium DMEM/F12 culture medium cooled on ice. The DRGs were completely shredded and digested with 0.25% trypsin and 0.1% type‐4 collagenase. followed by mixing and incubation at 37°C in a humidified atmosphere comprising 5% CO_2_ for 30 minutes to dissolve the tissue and release single cells. The process was assessed every 5 minutes, with gentle shaking to improve the mixing of the tissue pieces and the digestion solution. A fivefold volume of DMEM/F12 medium with 20% FBS was added to the centrifuge tube and allowed to equilibrate for 5 minutes without agitation to stop the digestion. Then, the cells were centrifuged at 300 × *g* for 5 minutes and resuspended in neurobasal serum‐free medium with B27 and 1% penicillin/streptomycin solution added. The cells were plated in 6‐well plates at 1 × 10^4^. After 24 hours, the medium was exchanged again, and a solution comprising 5 µmol/L cytarabine (MedChemExpress, Monmouth Junction, NJ), B27, 1% penicillin/streptomycin solution, and Neurobasal serum‐free medium was added. This medium was exchanged for normal culture medium after another 48 h.

### 2.4 Macrophage cultivation

Six‐ to eight‐week‐old female BALB/c mice were killed by administration of an anaesthetic overdose (0.5 mL/10 g 5% chloral hydrate) and placed in a 70% ethanol solution for 10 min. The complete femur and tibia were removed and the metaphyses of these bones were opened. The filtrate was repeatedly agitated to obtain a well‐mixed suspension. A 200 screen mesh (YA0949; Solarbio, China) was used to filter the obtained liquid, which was collected in a 15‐mL centrifuge tube, to which a threefold volume of red‐blood‐cell lysis solution was added and incubated at 37°C for 10 min to obtain complete lysis of erythrocytes. The resulting mixture in the 15‐mL centrifuge tube was centrifuged at 300 × *g* for 5 minutes to discard the supernatant. The pellet containing the cells was gently resuspended in preconditioned DMEM medium with 10% FBS, 1% penicillin/streptomycin solution and 10 ng/mL macrophage colony‐stimulating factor (M‐CSF; Sino Biological Inc, Beijing, China). The cell suspension was seeded into 35‐mm‐diameter culture plates at 1 × 10^6^, and the medium was changed every 2 days for 7 days until the cell cultures matured.

### 2.5 Experimental grouping and establishment of an in vitro irradiation model

Macrophages were randomly divided into six groups according to the treatments: a normal macrophage group (CON), an M1 macrophage group (M1), an M1 macrophage with LLLT group (M1+LLLT), a normal macrophage with inhibitor group (CON+in), an M1 macrophage with inhibitor group (M1+in), and an M1 macrophage with inhibitor and LLLT group (M1+LLLT+in). The number of cells was equal in each group.

CON group: Macrophages received normal medium.

M1 group: Macrophages received medium with the addition of 100 ng/mL lipopolysaccharide (LPS; Sigma‐Aldrich, St. Louis, MO) and 20 ng/mL interferon‐γ (IFN‐γ; Peprotech, Rocky Hill, NJ).

M1+LLLT group: Macrophages received medium with the addition of 100 ng/mL LPS and 20 ng/mL IFN‐γ and were irradiated by laser.

CON+in group: Macrophages were cultured with medium containing H89 (MedChemExpress, Monmouth Junction, NJ), an analogue of PKA and classic inhibitor of the PKA‐CREB pathway, which was added to the macrophage culture medium 48 hours before maturation at a concentration of 10 nmol/L. After the macrophages matured, the cell culture medium was changed to normal medium.

M1+in group: Macrophages were cultured in the same way as in the CON+in group before maturation. Subsequently, the cell culture medium was changed to medium with 100 ng/mL LPS and 20 ng/mL IFN‐γ.

M1+LLLT+in group: Macrophages were cultured in the same way as in the CON+in group before maturation. Subsequently, the cell culture medium was changed to medium with 100 ng/mL LPS and 20 ng/mL IFN‐γ and laser irradiation was performed.

The M1+LLLT and M1+LLLT+in groups were irradiated in a black box by a GLORIA‐X150A 150 W infrared xenon light source (Zolix, Beijing, China), whereas the CON, CON+in, M1, and M1+in groups were placed in another black box without irradiation. The parameters of the in vitro irradiation model were as follows: wavelength, 810 nm; power density, 2 mW/cm^2^; illumination area, 4.5 cm^2^; and irradiation time, 440 s. This resulted in an energy gain of 4 J (2 mW/cm^2^ × 4.5 cm^2^ × 440 s).

### 2.6 Flow cytometry

Macrophages received medium with the addition of 100 ng/mL LPS and 20 ng/mL IFN‐γ for 48 h. After cell maturation, 0.25% trypsin with 0.02% EDTA (Gibco, Grand Island, USA) was added, and 12‐well were plates into the incubators for 10 minutes during digestion. DMEM with 10% FBS was added to terminate digestion, the cells were placed into a centrifuge at 300*g* × 5 minutes, and the supernatant was discarded. Cells were resuspended in flow cytometry liquid and blocked with 20 μL rat serum. PE‐F4/80 antibody, positive control antibody and FITC‐CD86 (BioLegend, San Diego, CA) were added at 1:200. The samples were mixed thoroughly and incubated at 4°C for 1 hour. Then, they were washed with flow cytometry liquid twice and assessed on a flow cytometry machine (Beckman Coulter, CA, USA).

### 2.7 Cultivation of DRG neurons in macrophage‐conditioned medium

Forty‐eight hours after irradiation, the culture medium was removed from all groups and centrifuged at 10,000 × *g* for 10 minutes to remove cells and debris, yielding the conditioned medium, which was added to the culture dishes with purified DRG neurons. After another 48 hours, cell staining was conducted.

### 2.8 RT‐qPCR

Twenty‐four hours after the LLLT, the OMEGA RNA extraction kit (BioLegend, San Diego, CA) was used according to the manufacturer's protocol to extract the total RNA. The RNA obtained was reverse‐transcribed using PrimeScript RT Master Mix (TaKaRa Bio Inc, Kusatsu, Japan) according to the manufacturer's protocol. The beta‐actin housekeeping gene was used for normalization of gene expression by parallel amplification. The relative expression levels of the target mRNA were calculated. PCR primer sequences are listed in Table 1.

**Table 1:** Primers used in this study

Target gene/primer name	Primer sequence
BDNF	5’‐GGGTGAAACAAAGTGGCTGT‐3’(forward)
	5’‐ATGTTGTCAAACGGCACAAA‐3’(reverse)
LIF	5’‐TCAACTGGCTCAACTCAACG‐3’(forward)
	5’‐ACCATCCGATACAGCTCGAC‐3’(reverse)
NGF	5’‐CCTGCCAGAGTCCTTTTCTG‐3’ (forward)
	5’‐GGTTCAGGCCACAAAGTGTT‐3’ (reverse)
CNTF	5’‐CACCCCAACTGAAGGTGACT‐3’ (forward)
	5’‐ACCTTCAAGCCCCATAGCTT‐3’ (reverse)
β‐Actin	5’‐AGAAGGACTCCTATGTGGGTGA‐3’(forward)
	5’‐CATGAGCTGGGTCATCTTTTCA‐3’(reverse)

### 2.9 Western blot analysis

Forty‐eight hours after LLLT irradiation, the medium was removed from all the experimental groups, and the cells were washed with PBS, followed by the addition of 100‐150 µL RIPA buffer with phosphatase inhibitor and digestion for 5 minutes at 4°C. A cell scraper was used to completely harvest the protein from the bottom of the culture plate, which was transferred to a 1.5‐mL centrifuge tube and centrifuged at 4°C and 12 000 rpm for 5 min. The DNA in the centrifuge tube was discarded. A protein assay kit (Thermo Fisher Scientific, Waltham, MA) with bovine serum albumin (BSA; Thermo Fisher Scientific) as the protein standard was used to determine the protein concentration. Then, 20 µg of total protein was added to each well, and electrophoresis and transfer were performed. The membrane was blocked and incubated at 4°C overnight with the following primary antibodies (all 1:1000, Cell Signaling Technology, Danvers, MA): rabbit anti‐PKA, rabbit anti‐CREB, rabbit anti‐phosphorylated CREB (anti‐p‐CREB), rabbit anti‐ERK, rabbit anti‐p‐ERK, rabbit anti‐iNOS, rabbit anti‐ARG1 and mouse anti‐β‐tubulin. Then, the membrane was washed and incubated at room temperature for 1 hour with secondary antibodies (both 1:3000, GeneTex, Irvine, CA): HRP‐conjugated goat anti‐rabbit secondary antibody and HRP‐conjugated rabbit anti‐mouse secondary antibody. Trident femto Western HRP Substrate (GeneTex) was used to develop the membranes, which were imaged and analysed using an Amersham Imager 600 Series image station (Amersham, Little Chalfont, UK). ImageJ software (National Institutes of Health, Bethesda, MD) was used for densitometric analysis of the bands. The optical densities of the standard protein band and the internal standard (tubulin) were used to normalize the data and obtain the relative protein expression levels of all analysed proteins.

### 2.10 Immunofluorescence staining

The cells were transferred into immunofluorescence plates (SB‐Shifix20; Shikhar Biotech, Lalitpur, Nepal); washed with PBS three times, 5 minutes each; permeabilized using 0.3% Triton X‐100 for a total of 15 minutes; and blocked with 1% FBS at room temperature for 1 hour. The samples were incubated at room temperature overnight with mouse monoclonal anti‐βIII‐tubulin, anti‐F4/80 or rabbit polyclonal anti‐iNOS or anti‐NeuN (both 1:500, Abcam, Cambridge, MA). After the primary antibody was removed, the samples were washed with PBS three times, 5 minutes each, followed by incubation in the dark at room temperature for 40 minues with either Alexa Fluor 594‐conjugated donkey anti‐mouse secondary antibody or Alexa Fluor 488‐conjugated donkey anti‐rabbit secondary antibody (both 1:1500, Abcam).

### 2.11 ELISA

Forty‐eight hours after LLLT irradiation, the culture supernatants were collected, centrifuged to remove any floating cells and prepared as samples for ELISA, together with BDNF, NGF, CNTF and LIF standards, according to the instructions of the ELISA kits (BOSTER, Wuhan, China). After completion of the reaction, the absorbance of the samples and standards was measured at 450 nm using a microplate reader. After the absorbance curves of the standards were plotted, the concentrations of BDNF, NGF, CNTF and LIF were calculated for each sample.

### 2.12 Data analysis

Bar charts were rendered using GraphPad Prism 7.0 (GraphPad Software). Axon length analysis was conducted using ImageJ software. Statistical analysis was conducted using SPSS 19.0 (IBM Corp.). Each experiment was independently repeated five times, with the mean values used as the results. The data are expressed as the mean ± standard error of the mean. The results were initially tested for normality using the *F* test. Parametric data were compared using one‐way analysis of variance (ANOVA). Nonparametric data were compared using Tukey's test. Sidak's test was used to compare each group with the corresponding group that received the inhibitor. *P* < .05 was considered to indicate statistical significance.

## 3 RESULTS

### 3.1 Identification of macrophages in vitro

**Figure 1:**
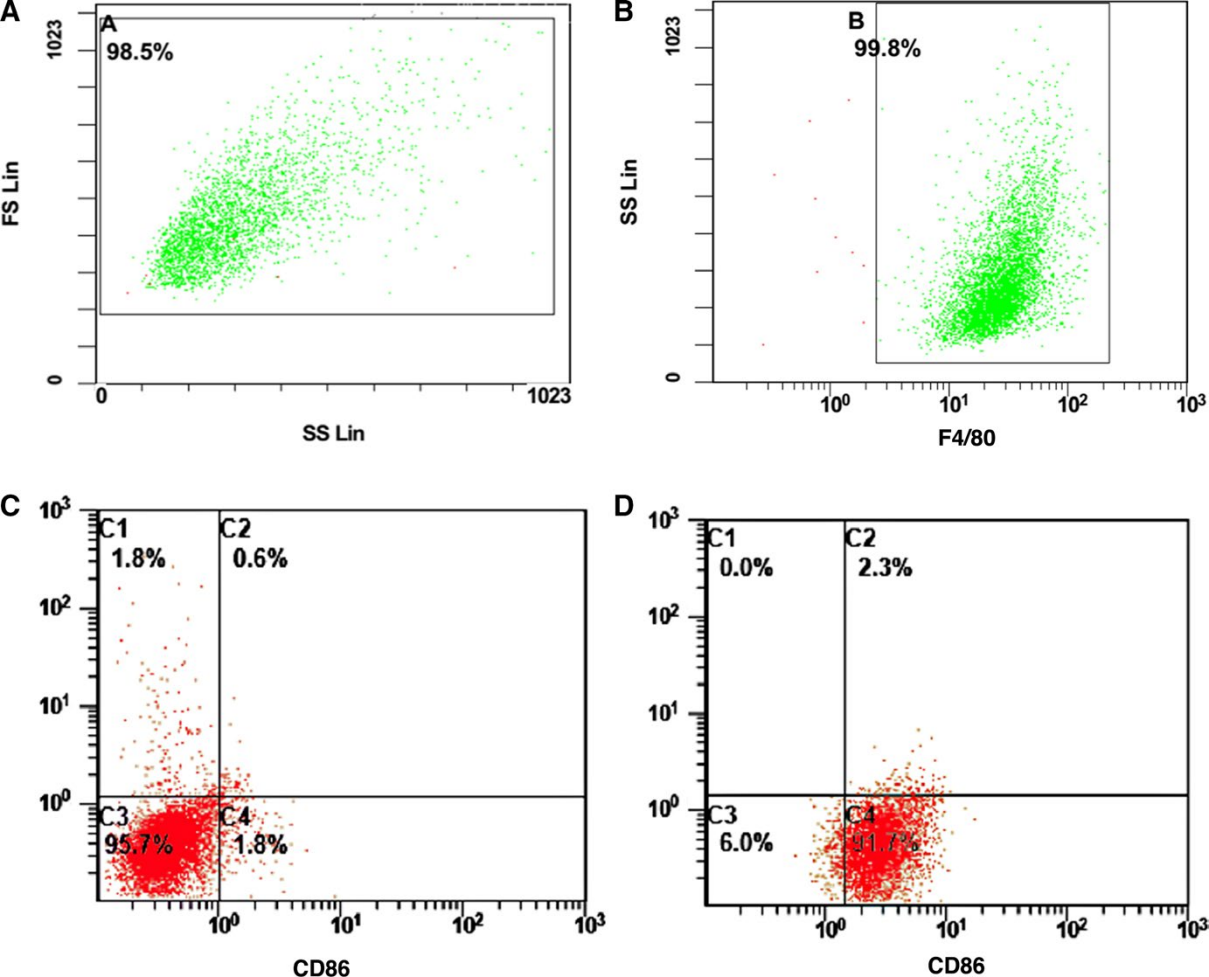
Flow cytometry was used to identify the primary cultured macrophages and the polarization of M1 macrophages. A and B, The ratio of F4/80^+^. Flow cytometry was used to identify. C, The ratio of CD86^+^, F4/80^+^ M1‐type macrophages was changed before adding LPS and IFN‐γ. D, The ratio of CD86^+^, F4/80^+^ M1‐type macrophages was changed after adding LPS and IFN‐γ

The results from flow cytometry showed that the positive ratio of F4/80 was 99.8% (region B), indicating that macrophages were cultured successfully (Figure 1A,B).

### 3.2 Identification of M1‐type macrophages in vitro

The results from flow cytometry showed that after addition of LPS and IFN‐γ, the ratio of CD86^+^, F4/80^+^ M1‐type macrophages increased from 1.8% to 91.7% (C4 quadrant), indicating successful polarization of macrophages to M1‐type macrophages (Figure 1C,D).

**Figure 2:**
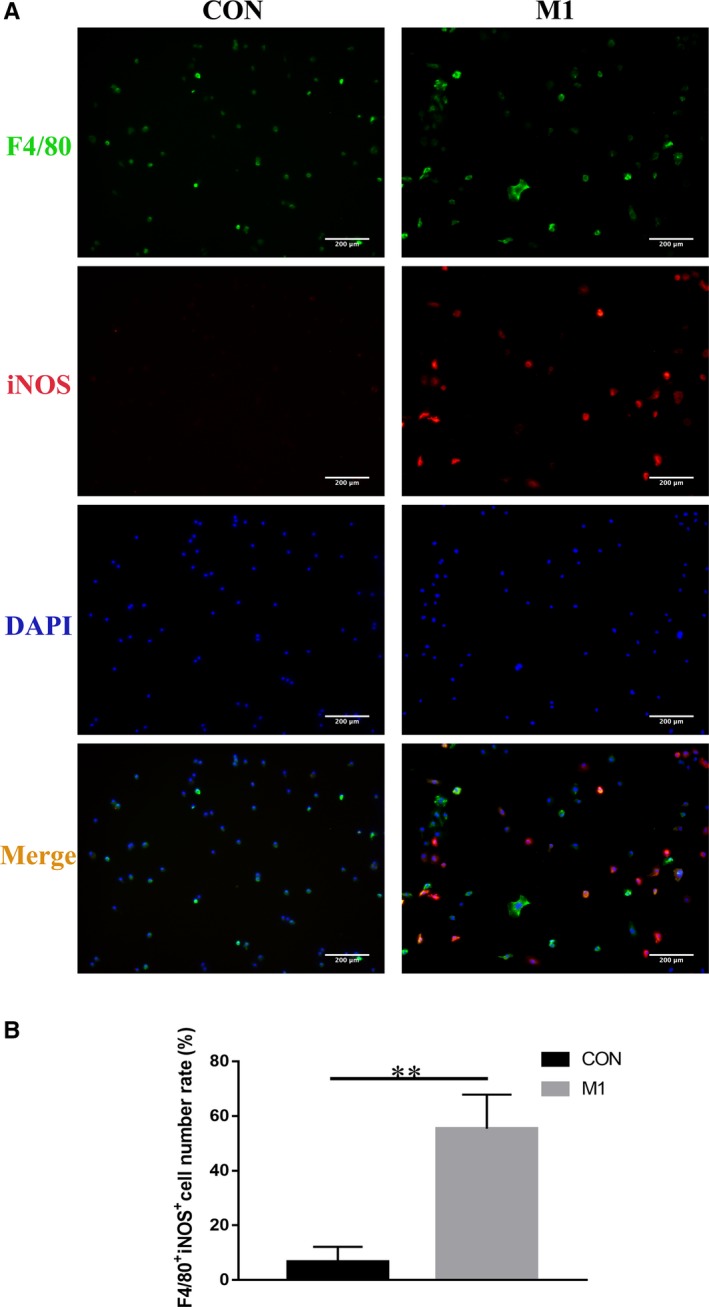
A, Immunofluorescence was used to identify the polarization of M1‐type macrophages. F4/80 is a macrophage marker, and iNOS is an M1 macrophage marker. B, The ratio of F4/80^+^iNOS^+^ cells in the M1 group was significantly improved compared with the CON group. **P < .01

The immunofluorescence results showed that after addition of LPS and IFN‐γ, the ratio of F4/80 ^+^iNOS^+^ macrophages in the M1 group (55.3 ± 8.8%) was significantly higher than that in the CON group (6.6 ± 3.9%) (*P* < .01) (Figure 2).

### 3.3 The influence of LLLT on M1 macrophages in vitro

**Figure 3:**
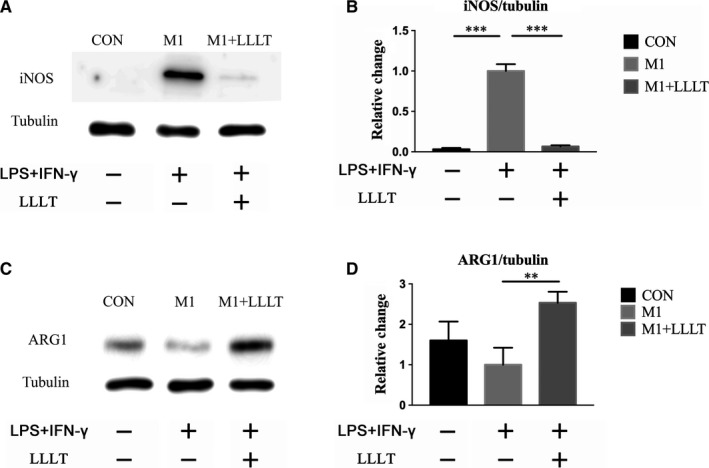
Western blot was used to investigate the expression levels of iNOS and ARG‐1. A and B, Expression level of iNOS. C and D, Expression level of ARG‐1. *P < .05, **P < .01, ***P < .001 compared with the M1 group

Compared with that of the M1 group, the iNOS expression was extremely low in the CON group and markedly reduced in the M1+LLLT group (both *P* < .001) (Figure 3A,B).

The ARG‐1 content of the CON group was higher than that of the M1 group, but the difference was not significant. Further comparison revealed that ARG‐1 expression was significantly increased in the M1+LLLT group compared with the M1 group (*P* < .01) (Figure 3C,D).

### 3.4 Influence of conditioned medium from M1 macrophages irradiated by LLLT on the axonal growth of DRG neurons

**Figure 4:**
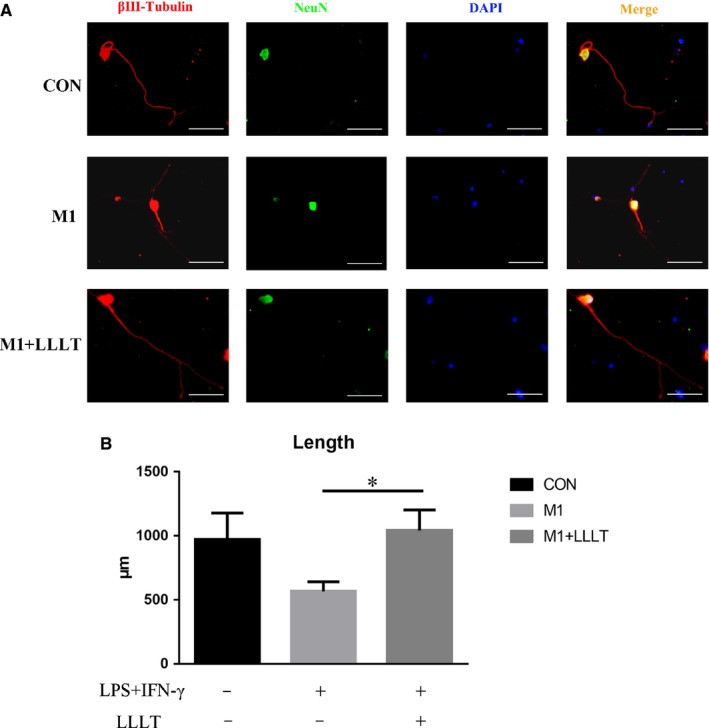
A, Forty‐eight hours after irradiation, the supernatants were individually collected and added to DRG neurons; after 48 h, immunofluorescence staining was used to assess axonal length with βIII‐tubulin as an axonal marker and NeuN as a neuronal body marker. B, The M1+LLLT group showed significantly improved neuronal growth compared with the M1 group. Bar = 200 μm. *P < .05 compared with the M1 group

The M1+LLLT group had significantly better neuronal growth than the M1 group (*P* < .05). Compared with that of the CON group, the length of the DRG axons was increased in the M1+LLLT group, while the length was shorter in the M1 group, but the differences were not significant (Table 2, Figure 4A,B).

**Table 2:** DRG axon length

Group	CON	M1	M1+LLLT
Length (μm)	971.88 ± 159.59	565.59 ± 58.02	1042.50 ± 123.49

Group	CON+in	M1+in	M1+LLLT+in
Length (μm)	463.23 ± 65.33	465.57 ± 77.21	601.54 ± 110.20

### 3.5 The influence of LLLT on the expression of neurotrophic factors by M1 macrophages

**Figure 5:**
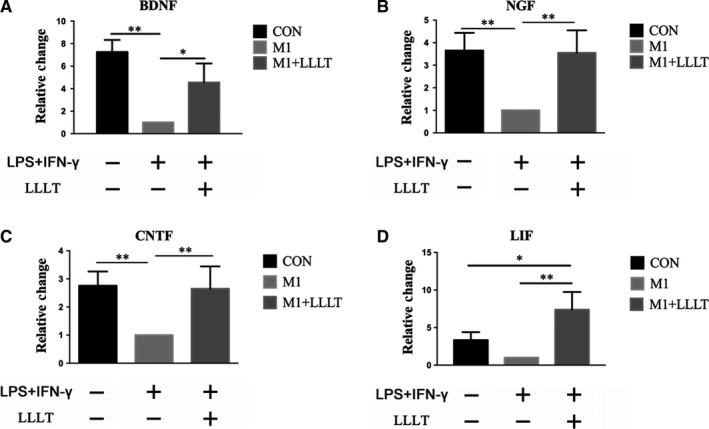
Twenty‐four hours after irradiation, RT‐qPCR was used to investigate the mRNA levels of BDNF, NGF, CNTF and LIF. The mRNA levels of the four investigated neurotrophic factors were increased in the M1+LLLT group compared with the M1 group. A, mRNA level of BDNF. B, mRNA level of NGF. C, mRNA level of CNTF. D, mRNA level of LIF. *P < .05, **P < .01, ***P < .001 compared with the M1 group

The mRNA levels of BDNF, NGF and CNTF were all significantly reduced in the M1 group compared with the CON group (*P* < .01). LIF was not significantly reduced (Figure 5A‐D). Furthermore, the mRNA levels of the neurotrophic factors were significantly increased in the M1+LLLT group compared with the M1 group (*P* < .05 for BDNF; *P* < .01 for NGF, CNTF and LIF). Additionally, the mRNA level of LIF was increased in the M1+LLLT group compared with the CON group (*P* < .05) (Figure 5A‐D).

**Figure 6:**
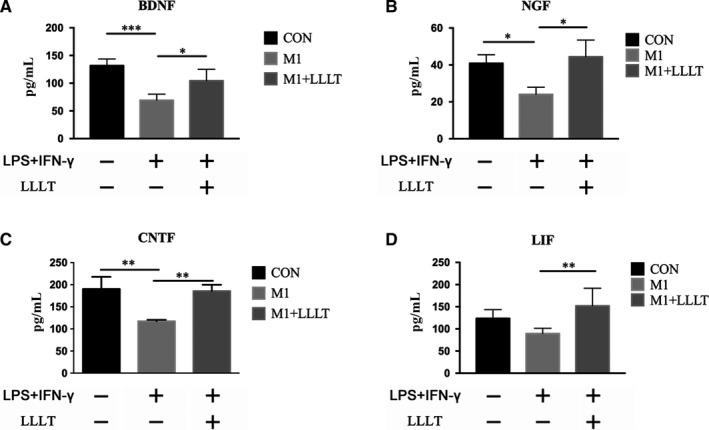
Forty‐eight hours after irradiation, ELISA was used to investigate the secretion quantities of BDNF, NGF, CNTF and LIF. The secretion quantities of the four investigated neurotrophic factors were increased in the M1+LLLT group compared with the M1 group. A, Secretion quantity of BDNF. B, Secretion quantity of NGF. C, Secretion quantity of CNTF. D, Secretion quantity of LIF. *P < .05, **P < .01, ***P < .001 compared with the M1 group (ANOVA)

The secreted protein levels of BDNF, NGF, CNTF and LIF were reduced to different extents in the M1 group compared with the CON group. The secretion of BDNF, NGF, CNTF and LIF was higher in the CON group than in the M1 group (Figure 6A‐D), with significant differences for all except LIF (BDNF, *P* < .001; NGF, *P* < .05; CNTF, *P* < .01). Furthermore, the secretion of the four investigated neurotrophic factors was significantly increased in the M1+LLLT group compared with the M1 group (BDNF, *P* < .05; NGF, *P* < .05; CNTF, *P* < .01; LIF, *P* < .01) (Table 3, Figure 6A‐D).

**Table 3:** Secretion of neurotrophic factors

Group		CON	M1	M1+LLLT
Target Protein (pg/mL)	BDNF	131.70 ± 10.44	69.33 ± 9.41	104.34 ± 17.91
	NGF	40.86 ± 3.80	24.06 ± 3.15	44.42 ± 7.40
	CNTF	190.30 ± 22.41	117.33 ± 2.54	185.70 ± 11.57
	LIF	123.64 ± 17.63	89.57 ± 10.46	151.97 ± 35.53

Group		CON+in	M1+in	M1+LLLT+in
Target protein (pg/mL)	BDNF	64.60 ± 7.00	53.13 ± 11.83	46.89 ± 11.61
	NGF	12.55 ± 0.62	16.36 ± 4.93	22.15 ± 12.43
	CNTF	98.016 ± 5.56	92.64 ± 1.44	96.15 ± 1.32
	LIF	87.62 ± 9.84	73.12 ± 11.37	85.91 ± 9.72

### 3.6 The influence of LLLT on neurotrophic secretion signalling by M1 macrophages

**Figure 7:**
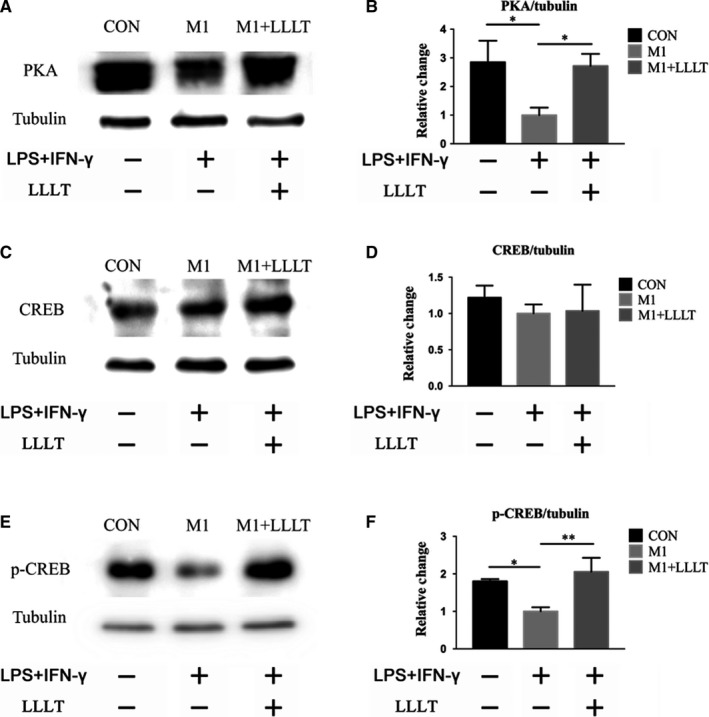
Influence of LLLT on the expression and phosphorylation of proteins in pathways related to the secretion of neurotrophic factors by M1 macrophages. Forty‐eight hours after the irradiation, Western blot was used to investigate the expression of PKA, CREB and p‐CREB. A and B, Expression level of PKA. C and D, Expression level of CREB. E and F, Expression level of p‐CREB. *P < .05, **P < .01, ***P < .001 compared with the M1 group

The expression of PKA was significantly higher in the CON group than in the M1 group (*P* < .05). The expression of PKA was also significantly higher in the M1+LLLT group than in the M1 group (*P* < .05) (Figure 7A,B). The levels of CREB were somewhat higher in both the CON and M1+LLLT groups than the M1 group, but the differences were not significant (Figure 7C,D). Further analysis of the expression level of p‐CREB revealed that CREB phosphorylation was significantly higher in the CON group than in the M1 group (*P* < .05). Furthermore, the p‐CREB levels were higher in the M1+LLLT group than in the M1 group (*P* < .01). There was no significant difference in the p‐CREB levels between the M1+LLLT and CON groups (Figure 7E,F).

Additionally, the expression levels of ERK and p‐ERK were investigated. The results did not indicate any significant difference in the levels of ERK between the two groups (Figure S1).

### 3.7 Effect of the PKA inhibitor H‐89 on the LLLT‐induced secretion of neurotrophic factors

**Figure 8:**
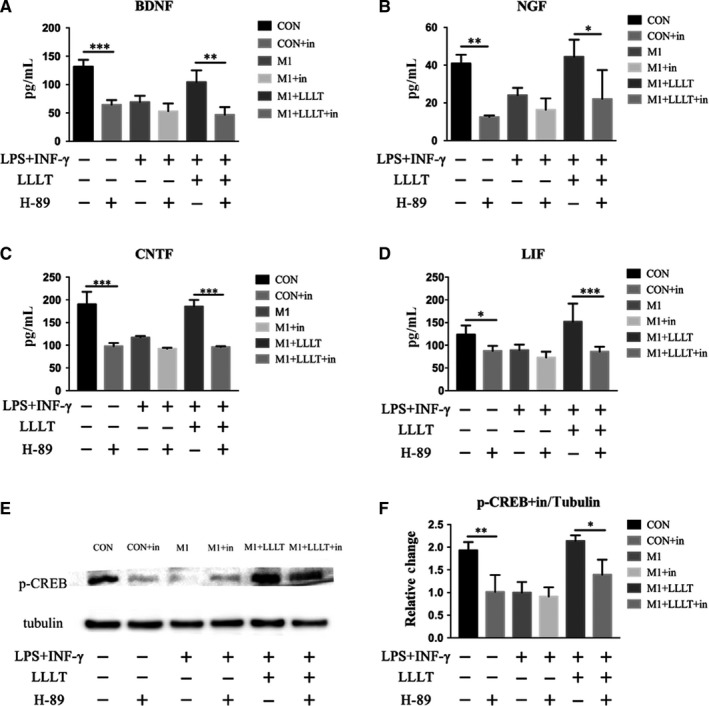
ELISA was used to investigate the secretion quantities of BDNF, NGF, CNTF and LIF. A, Secretion quantity of BDNF. B, Secretion quantity of NGF. C, Secretion quantity of CNTF. D, Secretion quantity of LIF. E and F, Influence of the PKA inhibitor H‐89 on the expression of p‐CREB in macrophages, examined using Western blot. *P < .05, **P < .01, ***P < .001. Each group was compared according to inhibitor presence

The secretion of neurotrophic factors was significantly higher in the CON group than in the CON+in group (BDNF, *P* < .001; NGF, *P* < .01; CNTF, *P* < .001; LIF, *P* < .05). Moreover, the secretion of all four neurotrophic factors was significantly higher in the M1+LLLT group than in the M1+LLLT+in group (BDNF, *P* < .01; NGF, *P* < .05; CNTF, *P* < .001; LIF, *P* < .001). However, there were no significant differences in the secretion of BDNF, NGF, CNTF or LIF between the M1 and M1+in groups (Table 3, Figure 8A‐D).

The p‐CREB levels of each group were compared with those of the corresponding group that received inhibitor. There were no significant differences between the M1 and M1+in groups. However, the p‐CREB levels were significantly higher in the CON group than in the CON+in group (*P* < .01), whereas those of the M1+LLLT group were higher than those of the M1+LLLT+in group (*P* < .05) (Figure 8E,F).

### 3.8 Effect of the PKA inhibitor H‐89 on the LLLT‐induced secretion of neurotrophic factors

The secretion of neurotrophic factors was significantly higher in the CON group than in the CON+in group (BDNF, *P* < .001; NGF, *P* < .01; CNTF, *P* < .001; LIF, *P* < .05). Moreover, the secretion of all four neurotrophic factors was significantly higher in the M1+LLLT group than in the M1+LLLT+in group (BDNF, *P* < .01; NGF, *P* < .05; CNTF, *P* < .001; LIF, *P* < .001). However, there were no significant differences in the secretion of BDNF, NGF, CNTF or LIF between the M1 and M1+in groups (Table 3, Figure 8A‐D).

The p‐CREB levels of each group were compared with those of the corresponding group that received inhibitor. There were no significant differences between the M1 and M1+in groups. However, the p‐CREB levels were significantly higher in the CON group than in the CON+in group (*P* < .01), whereas those of the M1+LLLT group were higher than those of the M1+LLLT+in group (*P* < .05) (Figure 8E,F).

### 3.9 Effect of the PKA inhibitor H‐89 on the axonal growth effect elicited by LLLT

**Figure 9:**
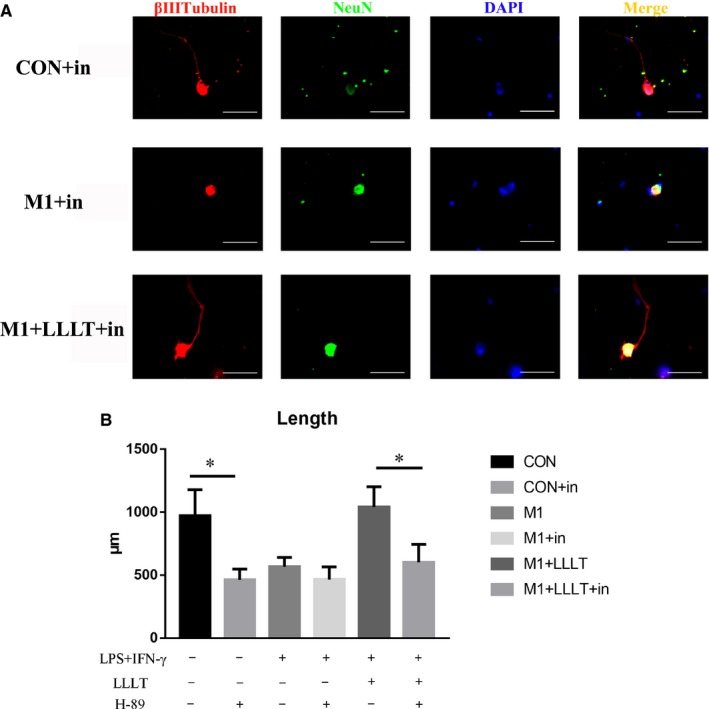
A, Immunofluorescence staining was used to analyse the length of axons, with βIII‐tubulin as an axonal marker and NeuN as a neuronal body marker. B, The axon length of each group was compared according to inhibitor presence. *P < .05, **P < .01, ***P < .001

Axonal growth was significantly greater in the CON group than in the CON+in group (*P* < .01), whereas that of the M1+LLLT group was also greater than that of the M1+LLLT+in group (*P* < .01) (Table 3). However, there were no significant differences between the M1 and M1+in groups (Figure 9).

## 4 DISCUSSION

After SCI, the formation of the lesion produces a physiological microenvironment that inhibits neuronal survival, blocks axon regrowth and leads to irreversible damage to the spinal cord.^14^ Macrophages play key roles in both time and space in the physiological microenvironment.^15^ Increasing numbers of studies have shown that macrophages have different polarization states in different environments. M1 macrophages promote the secretion of proinflammatory factors and are neurotoxic, whereas M2 macrophages can suppress the inflammatory reaction and promote axonal growth.^16^ Following SCI, M1 macrophages dominate the lesion microenvironment.^17^ In recent years, a number of researchers have pinpointed the polarization state of macrophages in the SCI lesion microenvironment and used different approaches to influence it. Liu et al 18 used extracts of *Mahonia oiwakensis* to promote the polarization of macrophages towards the M2 type, whereas Geng et al 19 used high‐pressure oxygen therapy to achieve the same effect and thus promote the healing of SCI.

In contrast, this study used LLLT, which is a widely clinically used noninvasive therapy that can suppress inflammation and promote recovery.^20^ Early studies have already discovered that LLLT can increase the production of IL‐4 and IL‐10 within the lesion site, down‐regulate the relative abundance of M1 macrophages and increase the number of M2 macrophages.^12^ To confirm the direct effect of LLLT on the regulation of M1 macrophages, we have innovatively established an in vitro LLLT‐M1 macrophage irradiation model. LPS and IFN‐γ were used to obtain M1 macrophages, which predominate in the SCI pathology environment. The M1 macrophages were irradiated, and different irradiation parameters (0‐10 J) were compared. The results showed that 4 J irradiation could significantly inhibit the inflammatory secretion of M1 macrophages.^21^ Compared with other studies on the relationship between LLLT and macrophages that used macrophage tumour cell lines,^22^ our study used primary macrophages, which are closer to the real environment in the spinal cord after injury. In addition, the selected illumination parameters were confirmed to have anti‐inflammatory effects in the early experiments, which laid the foundation for further research on its therapeutic effects and mechanisms in SCI. The results of the present study further confirmed that 810‐nm LLLT could significantly reduce the expression of the M1 macrophage marker iNOS in vitro, reducing it to levels close to those of nonstimulated macrophages. Moreover, the expression of the M2 macrophage marker ARG‐1 in the M1+LLLT group was increased compared with that of the M1 group. These results therefore demonstrated that LLLT can inhibit the expression of the M1 macrophage phenotype and promote their conversion to M2 macrophages.

Recent research has revealed that M2 macrophages can secrete many types of neurotrophic factors, including BDNF, NGF, CNTF and LIF.^23^, ^24^ Many researchers have attempted to improve the growth of axons by promoting the macrophage secretion of neurotrophic factors. Kwon et al 25 used the conditioning injury of peripheral neurons, which significantly increased the secretion of neurotrophic factors by macrophages and promoted the growth of DRG axons following SCI. Kuo et al 23 used peripheral nerve grafting and administration of acidic fibroblast growth factor (aFGF) to the lesion site, which boosted the secretion of BDNF by macrophages within the graft site. These studies have already demonstrated that promotion of the secretion of neurotrophic factors by macrophages after injury can improve the neuronal growth site microenvironment, ultimately enhancing the regrowth of axons with the aim of functional recovery. However, these methods are complicated and drugs are difficult to obtain, and whether LLLT can be directly applied to macrophages in the lesion site physiological environment to promote the secretion of neurotrophic factors remains unknown.

This study used various different methods to prove at multiple levels that LLLT can counteract the physiological microenvironment that inhibits the secretion of neurotrophic factors and positively influence macrophages to promote the mRNA expression and protein levels of BDNF, NGF, CNTF and LIF. When the culture supernatants of the differently treated macrophages were collected and used to produce conditioned medium for the growth of DRG neurons, immunofluorescence after 48 hours of culture in said medium showed better axonal growth in the M1+LLLT group than in the M1 group. This result indicated that LLLT at 810 nm promoted the expression of neurotrophic factors in M1 macrophages, which in turn improved their secretory features by counteracting their neurotoxic effects, improving the nerve growth environment and finally promoting the growth of DRG neurons.

There is abundant literature on the pathways involved in the secretion of neurotrophic factors, and CREB phosphorylation has been shown to have a key function in many of these processes. Consequently, promotion of the phosphorylation of CREB can boost the levels of many neurotrophic factors within the nervous system, including BDNF, NGF, CNTF and LIF. CREB phosphorylation is regulated by proteins from many upstream pathways, and changes in PKA, ERK and similar proteins can promote CREB phosphorylation and consequently influence the production of neurotrophic factors. Jana et al 26discovered that Na‐benzoate can increase PKA and thereby promote CREB phosphorylation, which in turn increased the levels of neurotrophic factors in the central nervous system. Luo et al 27confirmed that activation of the cAMP/PKA‐CREB pathway can increase the secretion of BDNF in hippocampal neurons and ultimately promote the recovery of learning and memory impairment in rats under chronic unpredictable mild stress. Wang et al 10demonstrated that Shuyu capsules can increase the phosphorylation of CREB by activating the ERK‐CREB signal transduction pathway, thereby increasing the secretion of neurotrophic factors in the central nervous system of rats. At the same time, studies have revealed that LLLT can also promote the secretion of neurotrophic factors. Yan et al 28showed that LLLT can increase the phosphorylation level of CREB by increasing the expression of ERK in dorsal root ganglia, which in turn promoted the levels of neuronal neurotrophic factors. However, the exact mechanism by which LLLT improves the secretion of neurotrophic factors in M1 macrophages remains to be elucidated.

In this study, an LLLT‐M1 irradiation model was established and used in combination with Western blot analysis to investigate the influence of LLLT at 810 nm on the secretion of a number of neurotrophic factors. At the same time, we investigated how LLLT increases PKA expression and CREB phosphorylation, as well as the mutual relationship between the increase in the secretion of neurotrophic factors and the PKA‐CREB pathway. H‐89 is a widely used highly selective inhibitor of PKA/p‐CREB, and we applied it to further investigate the role of this pathway in the effect of LLLT on M1 macrophages. The cell cultures were treated with the inhibitor 48 hours prior to LLLT, and the medium was changed either to normal macrophage medium or medium with LPS/IFN‐γ to prevent traces of the H‐89 from influencing the axon growth of DRG neurons in subsequent experiments using the conditioned medium. The results consistently demonstrated that LLLT can improve the growth of DRG axons by stimulating the PKA pathway of M1 macrophages, which in turn promoted CREB phosphorylation and the secretion of many different neurotrophic factors.

## 5 CONCLUSION

Through this study, we verified the hypothesis that LLLT can promote spinal cord repair and functional rehabilitation in rats with SCI. LLLT can inhibit the macrophages polarized to M1‐type, promote the expression of neurotrophic factors by M1 macrophages via PKA/CREB signalling and then promote the axonal growth of DRG neurons. This study adds further details on the mechanism of action of LLLT in SCI from a completely new point of view, laying a foundation for its eventual clinical application.

## CONFLICT OF INTEREST

The authors declared that they have no conflicts of interest to this work.

## AUTHOR'S CONTRIBUTION

JWZ, JKS, QZ, XFS and XYH wrote the manuscript and analysed the data; JWZ, JKS, QZ, ZWL and KL developed the LLLT‐macrophage irradiation model and performed the molecular biology experiments; JWZ, JKS, QZ, JWS, JXZ and LQ performed the immunofluorescence staining; ZW, XYH and TD designed and supervised the experiments.
