# Supplementary material for: Low‐level laser therapy 810‐nm up‐regulates macrophage secretion of neurotrophic factors via PKA‐CREB and promotes neuronal axon regeneration in vitro
Source: J Cell Mol Med. 2019 Oct 31;24(1):476–87. doi: 10.1111/jcmm.14756 (PMC6933332; doi:10.1111/jcmm.14756)
Supplement: Supplementary file 1 [file JCMM-24-476-s001.docx]

Supporting Information

**Figure S1:**


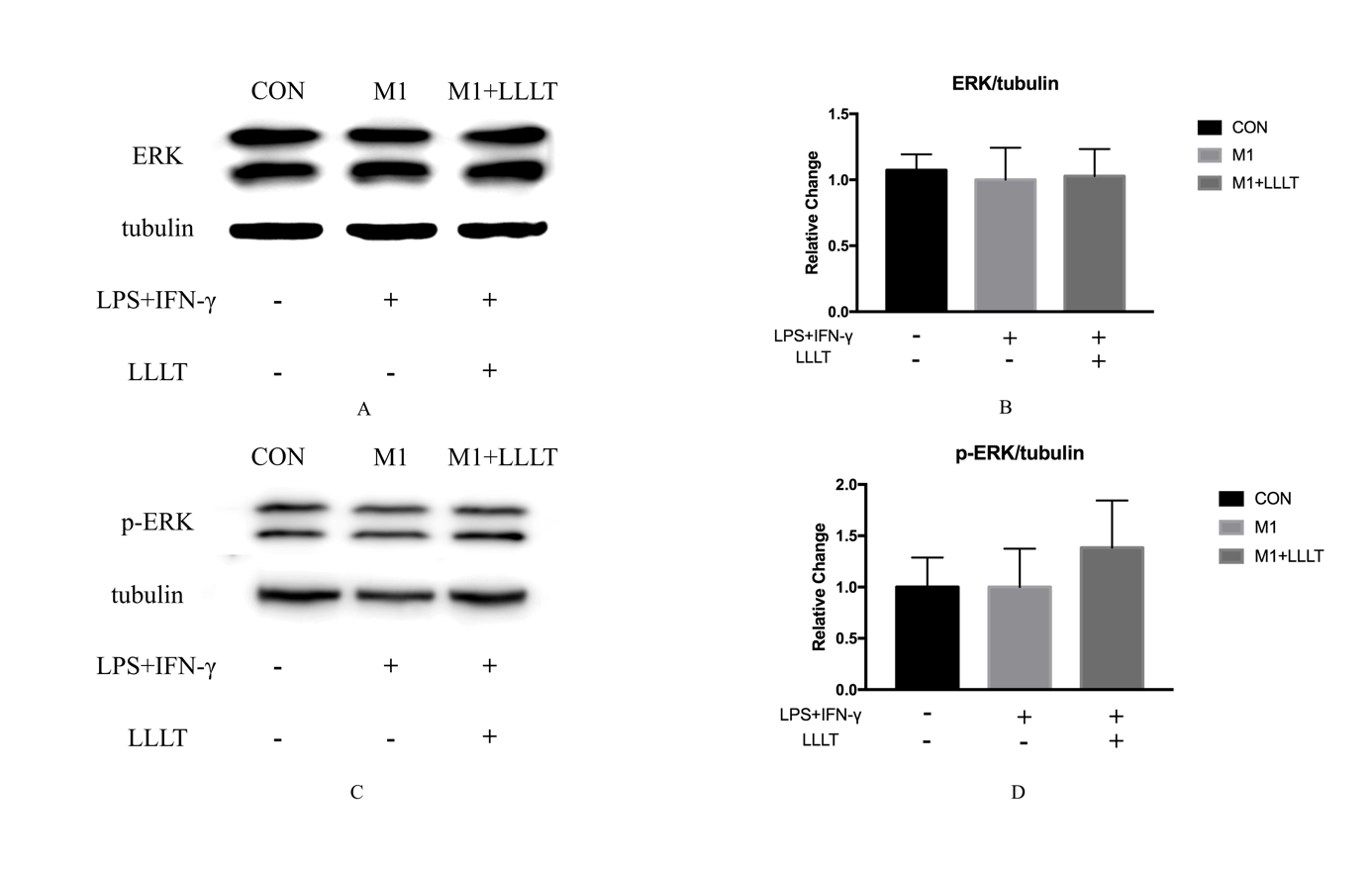


Figure S1: Influence of LLLT on the expression and phosphorylation of proteins in pathways related to the secretion of neurotrophic factors by M1 macrophages. Forty-eight hours after the irradiation, western blot was used to investigate the expression of ERK (A, B) and p-ERK (C, D). The results did not indicate any significant difference between the M1 and M1+LLLT groups in the levels of either of the two ERK forms.
